# A Table of Diseases, Arranged According to the Brunonian System

**Published:** 1799-06

**Authors:** 


### NOSOLOGY.

*Divisone delle malattie fatte secondo principi del sistema de Brown.—A Table of Diseases, arranged according to the Brunonian System.* By VALERIANO-LUIGI BRERA, Professor of Medicine at Pavia, 8vo. 40 pp. with a plate. *Pavia.*

The system of Brown appears to have met with more zealous advocates in in Italy, than it has done in France. This little tract is dedicated to Dr. Weikard, physician at Hailbron, one of the most ardent defenders the system has found in Germany, and is accompanied by tables of diseases according to Brown's plan. These tables are an improvement on those of Lynch, which were some time since added to the "Elements of Medicine" and consist of forty-five paragraphs, which Brera has explained by a "Dissertation."

The Brunonian system is too well known to professional men to need any explanation here. The tables, however, in the present work, will be found more perfect than any hitherto published, and well calculated for those who wish to compare other nosological systems with that of Brown.

